# Effect of gastrointestinal resection on sunitinib exposure in patients with GIST

**DOI:** 10.1186/1471-2407-14-575

**Published:** 2014-08-08

**Authors:** Djoeke de Wit, Nielka P van Erp, Reza Khosravan, Robin Wiltshire, Randy Allred, George D Demetri, Henk-Jan Guchelaar, Hans Gelderblom

**Affiliations:** Department of Clinical Pharmacy & Toxicology, Leiden University Medical Center, Leiden, The Netherlands; Department of Clinical Pharmacy, Radboud University Medical Center, Route 864, Postbus 9101, 6500 HB Nijmegen, The Netherlands; Pfizer Inc, San Diego, CA USA; Pfizer Inc, Tadworth, Surrey, England; Department of Medical Oncology/Solid Tumor Oncology, Ludwig Center at Dana-Farber Cancer Institute and Harvard Medical School, Boston, Massachusetts USA; Department of Clinical Oncology, Leiden University Medical Center, Leiden, The Netherlands

**Keywords:** Sunitinib, Exposure, Gastrointestinal resection, GIST

## Abstract

**Background:**

GIST patients often undergo GI-surgery. Previous studies have shown that imatinib and nilotinib exposures were decreased in GIST patients with prior major gastrectomy. We investigated whether major gastrectomy influences the exposure to sunitinib and its active metabolite SU12662.

**Methods:**

Pharmacokinetic data from 305 GIST patients included in 4 phase I-III trials were analyzed. Patients were subdivided into 6 groups according to their prior GI-surgery. Apparent clearance (CL/F) and dose-corrected steady-state plasma exposures (AUC_24,ss_) of sunitinib and SU12662 were estimated using a population PK approach. ANCOVA was performed to test for differences in AUC_24,ss_ and CL/F between each surgery subgroup and controls.

**Results:**

Major gastrectomy did not influence sunitinib or SU12662 exposure. The geometric mean of sunitinib and SU12662 AUC_24,ss_ was decreased by 21% and 28% in patients with both gastrectomy and small bowel resection (n = 8) compared to controls (n = 63) for sunitinib (931 ng*hr/mL (95%-CI; 676–1283) versus 1177 ng*hr/mL (95%-CI; 1097–1263); p < 0.05) and SU12662 (354 ng*hr/mL (95%-CI; 174–720) versus 492 ng*hr/mL (95%-CI; 435–555); p < 0.05). No significant differences in exposure were observed in each of the other subgroups versus controls.

**Conclusion:**

In contrast to previous results for imatinib and nilotinib, gastrectomy alone does not influence sunitinib or SU12662 exposure. This should be taken into account for the treatment of gastrectomized GIST patients with TKIs. In patients who had undergone both gastrectomy and small bowel resection, sunitinib and SU12662 exposures are significantly, although clinically not relevantly, decreased.

## Background

Gastrointestinal stromal tumors (GISTs) are the most common sarcoma of the gastrointestinal (GI) tract and highly resistant to conventional chemotherapy [1]. In 2001, imatinib was registered as first-line therapy for patients with primary unresectable and/or metastatic GIST [2, 3]. Thereafter in 2006, sunitinib was approved as second-line treatment for patients intolerant or refractory to imatinib therapy [4]. Recently, regorafenib was approved by the U.S. FDA as third-line therapy for GIST after failure of imatinib and sunitinib [5]. With the introduction of imatinib, sunitinib and regorafenib, survival of patients with metastatic GIST has substantially improved [4–6].

Imatinib C_trough_ levels and total sunitinib exposure have been reported to correlate with treatment benefit in patients with GIST [7, 8]. However, GIST patients often have an altered GI tract due to either resection of the primary tumor or subsequent surgery for recurrence and/or metastasis. Whether these alterations influence drug absorption and thus exposure and clinical outcome of treatment, depends on the physicochemical properties of the oral tyrosine kinase inhibitor given (Table 1).

**Table 1:** **Physicochemical properties of imatinib, nilotinib and sunitinib**

Drug	MW (g/mol)	pKa	Solubility	BCS class	Reference
Imatinib	493.60	7.7	Freely soluble (100–1000 mg/ml) up to pH 5.5. Solubility declines at higher pH; lowest solubility is 1 mg/ml.	II	[11]
Nilotinib	565.98	2.1 and 5.4	Slightly soluble (1–10 mg/ml) at pH 1.0, very slightly soluble (0.1-1 mg/ml) in water, at pH 3.0 and 3.0. Practically insoluble (<0.1 mg/ml) in buffer solutions of pH ≥ 4.5	IV	[12]
Sunitinib	398.47	9.0	25 mg/ml at pH 1.2-6.8. Solubility reduces at pH ≥ 6.8	IV	[16]

*Abbreviation*: *BCS* Biopharmaceutics Classification System, *MW* molecular weight.

A cross-sectional study in GIST patients treated with imatinib revealed that C_trough_ levels were significantly lower in patients that previously had a major gastrectomy compared to patients without gastric surgery [9]. Comparable results, relating decreased plasma drug exposures with prior major gastrectomy, were seen for GIST patients treated with nilotinib [10]. Since the solubility of imatinib and nilotinib rapidly declines above pH 5.5 and 4.5 respectively, it is suggested that in gastrectomized patients a decreased acid secretion may contribute to a decreased solubility and thereby decreased absorption of both TKIs [9–12]. Each segment of the gastrointestinal tract has its own characteristic pH level; acidity declines over the GI tract from the stomach (pH 1–3) to the small intestine (pH 5–7) and the colon (pH 7–8) [13, 14]. For imatinib and nilotinib solubility and absorption therefore rapidly decreases after the stomach [15]. This is further supported by the relative short time to reach maximum plasma concentration (T_max_) for these drugs; 2–4 hours for imatinib and 3 hours for nilotinib [11, 12]. Hence, due to the physicochemical properties of imatinib and nilotinib, the stomach is essential for dissolution and absorption of these TKIs.

For sunitinib however, solubility does not decline until pH 6.8 [16]. This makes in theory the involvement of the stomach less critical for dissolution and absorption of sunitinib. This is further supported by the relative broad surface over which sunitinib is absorbed from the GI-tract, reflected by a long time to reach maximum plasma concentration of sunitinib, e.g. 6–12 hours [16]. We postulated that major gastrectomy would most likely not affect the exposure to sunitinib and its active metabolite SU12662. To confirm this hypothesis, we retrospectively investigated the effect of GI resections on sunitinib and SU12662 exposures in patients with GIST across 4 different phase I-III clinical trials. Our primary objective was to investigate the effect of major gastrectomy; secondary objectives were to determine the effect of other GI resections on sunitinib exposure.

## Methods

### Patient selection

A total of 635 patients were treated with sunitinib in 4 different phase I/II, II, or III clinical trials that investigated the safety, efficacy, and/or pharmacokinetics of sunitinib in patients with GIST [17–20]. Of these 635 patients, a total of 364 patients had pharmacokinetic (PK) samples available which were included in population pharmacokinetic analysis. Out of these 364 patients (for sunitinib total number of samples = 3394 and for SU12662 total number of samples = 3410), a total of 305 patients had comprehensive GI resection data available and were therefore eligible for the present analysis.

Inclusion criteria in these trials were: a histopathologically confirmed diagnosis of metastatic or unresectable GIST with progression on or toxicity of previous imatinib therapy; age > 18 years or between 20 to 75 years; adequate hematologic, renal, liver and cardiac function; an Eastern Cooperative Oncology Group (ECOG) performance status of 0 or 1; willingness and ability to comply with scheduled visits, treatment plans, laboratory test, and other study procedures.

Exclusion criteria in these trials were: current treatment in another clinical trial or ≤ 4 weeks prior to starting sunitinib, ≤ 2 weeks for imatinib therapy ; non recovery from acute toxic effect of previous chemotherapy or imatinib; a history of known brain metastases; any serious co-morbidity; and pregnancy or breastfeeding.

All studies were done in accordance with Good Clinical Practice and under the ethical principles established by the Declaration of Helsinki. Each protocol was reviewed and approved by the Institutional Review Board and informed consent was obtained from each patient. The sub-analysis on the existing dataset of Pfizer was requested by the non-Pfizer affiliated authors of this manuscript and was reviewed and granted by Pfizer.

### Sunitinib pharmacokinetic data collection and statistical analysis

Patients were treated with sunitinib in doses ranging from 25 mg to 75 mg once daily on 4/2 (4 weeks on treatment followed by 2 weeks off treatment), 2/1 (2 weeks on treatment followed by 1 week off treatment), 2/2 (2 weeks on treatment followed by 2 weeks off treatment) or CDD (continuous daily dosing) schedules. Blood samples for pharmacokinetic assessment of sunitinib and its active metabolite SU12662 were collected pre-dose or post-dose on different days with details provided in Table 2. Blood samples were collected in EDTA tubes and shortly after collection centrifuged at 4°C for 10 minutes at 3500 rpm. Plasma samples were separated and stored at −20°C or lower until shipped. Shipment of samples was on dry ice to Bioanalytical Systems Inc (West Lafayette, IN) where they were stored at −20°C or lower until assayed within the established stability window. For quantification a validated, sensitive and specific isocratic liquid chromatographic tandem mass spectrometric (LC-MS/MS) method in positive ionization mode was used [21].

**Table 2:** **Summary of characteristics of studies used for analyses**

Study number	Study design	Population (n)^a^	Dosing schedule: dose	Day(s) of PK sampling	Time point(s) of sampling	Reference(s)
RTKC-0511-013	Phase II	74	2/1: 50 mg	Days 1, 14, 28 (only 4/2) of Cycles 1, 2, and 3 (optional)	Pre-dose	[19]
			2/2: 25, 50, 75 mg		On 1^st^ day of Cycle 1 and on last day of Cycles 1 and 2: 0, 1, 4, 6, 8, 10, 12, 24, and 48 hr post-dose (10 and 12 hr optional)	
			4/2: 50 mg	Day 1 of Cycles 4, 5 (optional), and 6		
A6181004	Phase III	179	4/2: 50 mg	Days 1, 14, and 28 of Cycle 1; Days 1 and 28 of Cycles 2 and beyond	Pre-dose	[20]
A6181045	Phase I/II	33	4/2: 25, 50, 75 mg	Phase I: Days 1, 2, 7, 14, 21, and 28 of Cycle 1	Pre-dose	[18]
					On Days 1 and 28 of Cycle 1 (Phase I Only): 0, 1, 2, 4, 6, 8, 10, 24 (Only Day 28) and 48 (Only Day 28) hr post-dose	
				Phase II: Days 1, 14, and 28 of Cycles 1-4		
A6181047	Phase II	19	CDD: 37.5 mg	Day 1 of each cycle	Pre-dose	[17]

*Abbreviations*: *2/1* 2 weeks on treatment followed by 1 week off treatment, *2/2* 2 weeks on treatment followed by 2 weeks off treatment, *4/2* 4 weeks on treatment followed by 1 week off treatment, *CDD* continuous daily dosing, *PK* pharmacokinetics.

^a^Number of subjects from each study contributing to the ANCOVA.

All quantifiable plasma samples were included to develop population PK models for sunitinib and SU012662 using Nonlinear Mixed Effect Modeling software (NONMEM; version 7.1.2), following exclusion of plasma samples with inadequate dosing records and those identified to be extreme outliers (eg, 6 < Conditional Weighted Residual(CWRES) < −6). Sunitinib data were best described by a two-compartment model with first-order order absorption with a lag time and first-order elimination. Similarly, SU12662 data were best described by a two-compartment model with first-order formation without lag time and first-order elimination.

Patients were subdivided into 6 subgroups according to their previous GI surgery: 1) Major gastrectomy (defined as total or subtotal gastrectomy), 2) Partial gastrectomy, 3) Small bowel resection, 4) Both gastrectomy (either partial or (sub)total) and small bowel resection, 5) Colon resection, and 6) Controls with no prior surgery. Patients with uncertain or unclear defined GI resections (n = 59) were excluded from analysis.

Following population PK analyses, the individual post-hoc estimates for CL/F of sunitinib and SU12662 were used to calculate steady-state total plasma exposures (AUC_24,ss_) of sunitinib and SU12662 at 50 mg of sunitinib for each individual patient, by dividing the dose (i.e., 50 mg) by individual patient post-hoc CL/F estimate. Thereafter, an analysis of covariance (ANCOVA) on log transformed data was performed to test for significant differences in AUC_24,ss_ and CL/F of both sunitinib and SU12662 between each surgical subgroup and control. Covariates previously identified by Houk et al. which were initially included in the ANCOVA model were sex and race for sunitinib CL/F, and sex, race, body weight and ECOG performance status for SU12662 CL/F [22]. Within the ANCOVA models, Multiple Comparisons with Control (i.e., MCC) using Dunnett’s test were performed and significant increases in CL/F and decreases in AUC_24,ss_ were identified. For sunitinib and SU12662 CL/F the difference was considered statistically significant (p ≤ 0.05), if the 95% lower bound for the difference from controls on the log scale did not include zero. Conversely, for sunitinib and SU12662 AUC_24,ss_, if the 95% upper bound for the difference from the control, on the log scale, did not include zero, the difference was considered statistically significant (p ≤ 0.05). Subsequently, previously identified covariates which were not statistically significant (p > 0.05) within the ANCOVA model were later removed from the ANCOVA models for sunitinib and SU12662. The number of observation for each individual was added as an additional covariate to the ANCOVA models to make sure it did not affect the final ANCOVA models overall results and conclusions. All statistical analyses were performed using S-Plus Version 8.0 (TIBCO Software Inc., Palo Alto, USA). The population pharmacokinetic and statistical analysis on the existing dataset was done by Pfizer Inc. Independent reviewers, blinded to the PK and patient data and not related to Pfizer subdivided the included patients into 6 groups according to their previous GI surgery.

## Results

### Patient characteristics

A total of 305 patients had both population PK parameter estimates and comprehensive GI resection data available and were therefore included in the descriptive statistics presented as well as in the analysis of covariance (ANCOVA) models for sunitinib and SU12662. Of these patients, 45 underwent major gastrectomy (subgroup 1), 58 partial gastrectomy (subgroup 2), 118 small bowel resection (subgroup 3), 8 both gastrectomy and small bowel resection (subgroup 4) and 13 patients a colon resection (subgroup 5). Sixty-three patients served as controls and did not have any prior surgery (subgroup 6). Baseline characteristics including sex, age, bodyweight, ethnicity and ECOG performing status are shown in Table 3 per subgroup.

**Table 3:** **Patient characteristics for each past GI surgery subgroup**

	Major gastrectomy (n = 45)	Partial gastrectomy (n = 58 )	Small bowel resection (n = 118)	Combination of gastrectomy and small bowel resection (n = 8 )	Colon resection (n = 13)	Controls (n = 63)
**Sex, n (%)**						
**Male**	30 (66.7%)	35 (60.3%)	75 (63.6%)	6 (75%)	9 (69.2%)	40 (63.5%)
**Female**	15 (33.3%)	23 (39.7%)	43 (36.4%)	2 (25%)	4 (30.8%)	23 (36.5%)
Age (years)*	56 (36–77)	57 (28–79)	53 (23–81)	49 (45–54)	68 (50–84)	58 (36–84)
Bodyweight (kg)*	65 (40–100)	70 (39–121)	71 (40–140)	64 (45–139)	80 (56–114)	74 (44–137)
**Race, n (%)**						
**Non-Asian**	37 (82.2%)	52 (89.7%)	94 (79.7%)	7 (87.5%)	12 (92.3%)	59 (93.7%)
**Asian**	8 (17.8%)	6 (10.3%)	24 (20.3%)	1 (12.5%)	1 (7.7%)	4 (6.3%)
**ECOG performing status, n (%)**						
**≤ 1**	42 (93.3%)	58 (100%)	116 (98.3%)	8 (100%)	13 (100%)	62 (98.4%)
**≥ 2**	3 (6.7%)	0 (0%)	2 (1.7%)	0 (0%)	0 (0%)	1 (1.6%)

*Data are presented as median values with lower and upper limit.

### Effect of prior gastrointestinal surgery on sunitinib pharmacokinetics

**Figure 1:**
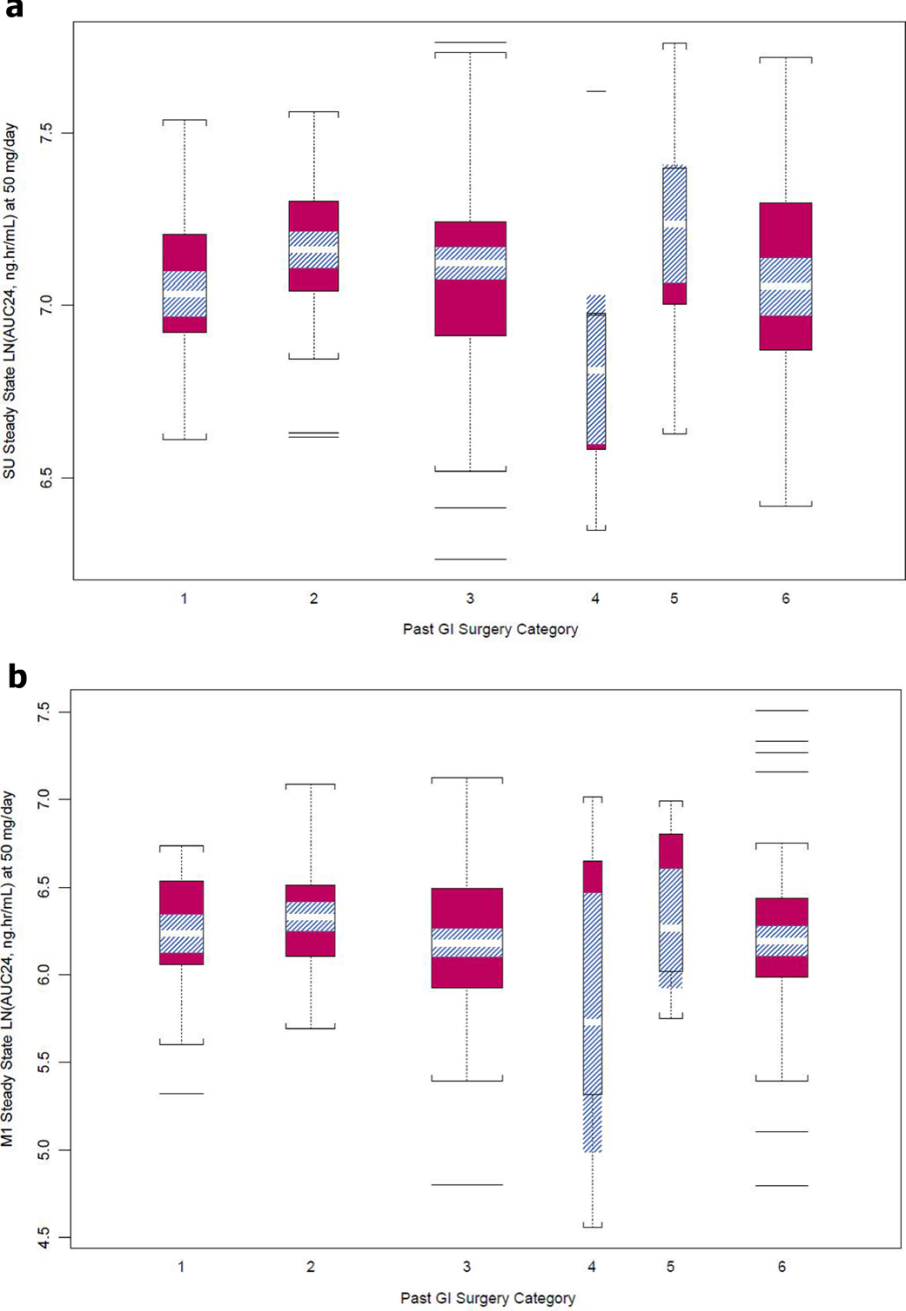
**Exposures in patients with different GI resections. a)** Sunitinib exposure. **b)** SU12662 exposure. Abbreviations: SU = sunitinib; M1 = SU12662. Legend: 1 = Major gastrectomy; 2 = Partial gastrectomy; 3 = Small bowel resection; 4 = Combination of gastrectomy and small bowel resection; 5 = Colon resection; 6 = Controls with no prior surgery.

Sunitinib and SU12662 apparent clearance (CL/F) was not increased in patients that previously had a major gastrectomy. Consequently, the geometric mean of sunitinib and SU12662 AUC_0-24hr_ were not decreased in patients with a major gastrectomy, compared to patients in the control subgroup for sunitinib (1171 ng*hr/mL versus 1177 ng*hr/mL; p > 0.05) and SU12662 (520 ng*hr/mL versus 492 ng*hr/mL p > 0.05) (Table 4 and Figure 1).

**Table 4:** **Sunitinib and SU12662 CL/F and AUC**_**24,ss**_ **estimates for each past GI surgery subgroup**

Parameter	Past GI surgery subgroup					
	Major gastrectomy (n = 45)	Partial gastrectomy (n = 58 )	Small bowel resection (n = 118)	Combination of gastrectomy and small bowel resection (n = 8 )	Colon resection (n = 13)	Controls (n = 63)
**Number of PK samples per subject, median (range)**	7 (1–32)	9 (1–38)	7 (1–35)	10 (2–35)	13 (2–30)	7 (1–37)
**Sunitinib**						
**AUC**_**24,ss**_ (ng*hr/mL)	1171 (1099–1248)	1294 (1228–1365)	1194 (1141–1250)	931 (676–1283)*	1325 (1109–1583)	1177 (1097–1263)
**CL/F (L/hr)**	42.7 (40.1 - 45.5)	38.6 (36.6 - 40.7)	41.9 (40.0 - 43.8)	53.7 ( 39.0 - 74.0)*	37.7 (31.6 - 45.1)	42.5 (39.6 - 45.6)
**SU12662**						
**AUC**_**24,ss**_ (ng*hr/mL)	520 (474–571)	567 (522–617)	492 (458–529)	354 (174–720)*	597 (457–779)	492 (435–555)
**CL/F (L/hr)**	20.2 (18.4 - 22.1)	18.5 (17.0 - 20.1)	21.4 (19.9 - 23.0)	29.7 (14.6 - 60.4)*	17.6 (13.5 - 23.0)	21.4 (18.9 - 24.1)

*Abbreviations*: *AUC*_*24,ss*_ Area Under the Concentration-time curve from time zero to 24 hours post-dose at steady state, *CL/F* apparent clearance, *PK* pharmacokinetic.

Data are presented as geometric mean (95% CI) unless stated otherwise. *Significantly different compared to controls (p < 0.05).

A significant increase in apparent clearance (CL/F) of sunitinib and SU12662 was seen in patients that had undergone both gastrectomy and small bowel resection relative to the controls. The geometric mean of CL/F for sunitinib and SU12662 was increased by 26% and 39% in subgroup 4, patients with both gastrectomy and small bowel resection, compared to those in the control subgroup for sunitinib (53.7 L/hr versus 42.5 L/hr; p ≤ 0.05) and for SU12662 (29.7 L/hr versus 21.4 L/hr; p ≤ 0.05), respectively. No statistically significant (p > 0.05) increases in apparent clearance for each of the other subgroups from controls were observed (Table 4 and Figure 1).

Consequently, a decreased total plasma exposure (AUC_24,ss_) to sunitinib and SU12662 was seen in patients that had undergone both gastrectomy and small bowel resection. The geometric mean of total plasma exposure (AUC_24,ss_) to sunitinib and SU12662 was 21% and 28% lower in subgroup 4, patients that underwent both gastrectomy and small bowel resection, compared to those in the control subgroup sunitinib (931 ng*hr/mL versus 1177 ng*hr/mL; p ≤ 0.05) and for SU12662 (354 ng*hr/mL versus 492 ng*hr/mL; p ≤ 0.05), respectively. No statistically significant (p > 0.05) decreases in total plasma exposures for each of the other subgroups compared to controls were observed (Table 4 and Figure 1).

## Discussion

This study shows that major gastrectomy did not affect sunitinib or SU12662 plasma exposures in patients with GIST. This is in contrast to prior data regarding the impact of gastrectomy on both imatinib and nilotinib exposure [9, 10]. Sunitinib and SU12662 exposures were however significantly decreased in patients who had previously undergone both gastrectomy and small bowel resection, although this observation was in a small subgroup of patients. All other types of GI resections studied showed no impact on sunitinib or SU12662 pharmacokinetics.

The results from this study support our hypothesis that the influence of GI resections on TKI exposure depends on two variables: the specific physicochemical properties of the TKI given and the part of the GI tract that has undergone resection. So although most TKIs exhibit pH-dependent solubility, small differences in their physicochemical properties (e.g. declined solubility in pH conditions higher than pH 5.5 for imatinib versus 6.8 for sunitinib) may cause great differences in the impact of gastrectomy on their GI solubility and absorption. In addition, the absorption characteristics of a drug under normal conditions [i.e. whether it is absorbed throughout the GI tract (e.g. sunitinib) or whether it is mainly absorbed through the stomach and the upper part of the small intestine (e.g. imatinib)] may affect the extent to which site specific GI resections can decrease the GI availability (F_gut_) and subsequently the bioavailability (F = F_gut_*F_hepatic_) of a drug. The finding that imatinib exposure is not affected by the co-administration of the proton pump inhibitor omeprazole somewhat contradicts our hypothesis considering reduced solubility [23]. However, 40 mg omeprazole only increases the gastric pH to 4.6 which is still an adequate level for imatinib to freely dissolve [24]. Major gastrectomy might result in a further rise in pH equally to that of the small intestines and this therefore could interfere with imatinib dissolution.

Currently, the approved and accepted first line treatment for GIST is imatinib [11]. The stomach is the most common primary site for GIST (~60%), and a proportion of these patients will therefore undergo major gastrectomy procedures prior to systemic imatinib therapy for metastasis [25]. Imatinib C_trough_ levels in ~80% of the gastrectomized patients were reported to be below 1100 ng/mL which has been correlated to shorter progression free survival (PFS) [7, 9]. In addition, increasing the imatinib dose might not result in an increased exposure due to the limited solubility of imatinib in a patient with limited gastric physiology. By measuring plasma concentrations in patients with prior major gastrectomy, a decreased exposure to imatinib could be identified early in treatment, prior to development of clinical drug failure. Sunitinib is currently approved and accepted as the second line treatment for GIST patients and also for sunitinib a relationship between systemic exposure and efficacy has been demonstrated before [8, 16]. The results from this present study show that sunitinib exposure is, in contrast to the results for imatinib, not decreased in gastrectomized patients. These findings should be taken into account for the treatment of gastrectomized GIST patients with TKIs. Hypothetically, gastrectomized patients have less and/or shorter treatment benefit from first-line imatinib therapy due to decreased imatinib plasma levels. Yet, these patients theoretically have a high chance of treatment benefit from second-line sunitinib therapy. However, further prospective research to investigate this hypothesis and whether there is a difference in clinical outcome between gastrectomized patients treated with imatinib or sunitinib is needed.

The results from this present analysis also show that patients who had undergone both gastrectomy and small bowel resection did have statistically significantly (p ≤ 0.05) lower sunitinib and SU12662 exposures, which is an extension of prior data showing such effects of combined surgery on plasma exposure of both imatinib and nilotinib [9, 10]. An effect of both gastrectomy and small bowel resection on the exposure to all three studied TKIs and other drugs is not surprising, since resections of large portions of the GI tract will significantly reduce the absorption surface available. Houk et al. showed that patients with GIST and a sunitinib AUC_ss_ > 600 ng*hr/mL had longer time to progression (TTP) and overall survival (OS) [8]. The patients with a combined gastrectomy and small bowel resection in our study had an average sunitinib exposure of 931 ng*hr/mL and none of the patients in this subgroup had a sunitinib exposure <600 ng*hr/mL. So although patients with both a gastrectomy and small bowel resection in this study had a statistically significant (p ≤ 0.05) decrease in sunitinib and SU12662 exposure, this decrease does not appear to be clinically relevant.

Hypothetically, the extent of small bowel resection will be critical for the remaining absorption surface and whether and to what extent sunitinib exposure is affected or not. Unfortunately, the length of resected intestine was not registered in the database used for this retrospective study, which limits the ability to analyze this variable. Measuring plasma concentrations of sunitinib could be suggested in patients that underwent an unknown or very large resection of the GI tract to identify those that do have a clinically relevant decreased sunitinib exposure. In clinics where the measurement of sunitinib plasma concentration is not feasible, an alternative and practical approach could be to gradually increase the dose based on the individual patient safety and tolerability. The relatively small number of patients who underwent a combined gastrectomy and small bowel resection (n = 8) can be considered as a limitation of this present study. Therefore, the results in this subgroup of patients should be interpreted with caution and need to be verified in a larger group of patients with extended GI resections.

It is generally assumed that for most weakly basic drugs, the dissolution process is often the rate-limiting step for absorption of these drugs from the GI tract. However, besides pH and physicochemical properties there are other variables within the GI tract that determine the rate and extent of dissolution including the fluid volume available for dissolution that is added in the stomach, gastric motility and the maximum dose strength. Also the maximum dose strength is rather different between imatinib, nilotinib and sunitinib. Imatinib and nilotinib are dosed at 400–800 mg a day compared to sunitinib which is dosed at 25–50 mg a day. This could be an additional explanation why sunitinib exposure is not influenced by gastrectomy whereas imatinib and nilotinib exposure are. Apparently, pH and dosage rather than fluid volume and gastric motility is of influence on the absorption of TKIs. An alternative pre-clinical explanation for the found differences is the removal due to gastrectomy of transporters that facilitate the gastric absorption of TKIs, whereby imatinib might depend more on this transporter for absorption than sunitinib does [26, 27].

## Conclusion

In conclusion, major gastrectomy alone does not influence exposure to sunitinib or its active metabolite SU12662, which is contrary to previous results for imatinib and nilotinib. This should be taken into account for the treatment of gastrectomized GIST patients with TKIs. Patients with a combined gastrectomy and small bowel resection had a statistically significantly, though clinically not relevant, decreased plasma exposure to sunitinib and SU12662 which in theory might depend on the length of intestine resected.
